# Challenges and considerations in interpreting the age-dependent benefit of neoadjuvant treatment for adenocarcinoma of the esophagus and gastroesophageal junction

**DOI:** 10.1097/JS9.0000000000001606

**Published:** 2024-05-10

**Authors:** Kexun Li, Hongchi Yan, Tengfei Ke

**Affiliations:** a Department of Thoracic Surgery I, Key Laboratory of Lung Cancer of Yunnan Province, Yunnan Cancer Hospital, The Third Affiliated Hospital of Kunming Medical University, Cancer Center of Yunnan Province; b Department of Radiology, Yunnan Cancer Hospital, The Third Affiliated Hospital of Kunming Medical University, Kunming; c Changning County Traditional Chinese Medicine Hospital, Yibin, China

*Dear Editor*,

We have carefully read the paper titled ‘Age-dependent benefit of neoadjuvant treatment in adenocarcinoma of the esophagus and gastroesophageal junction: a multicenter retrospective observational study of young versus old patients’ by Rompen *et al*.^1^ presents a well-executed multicenter retrospective cohort study. The authors utilized univariate and multivariate analyses to evaluate overall survival (OS) while considering confounding variables. Their objective was to provide evidence for the age-dependent use of neoadjuvant treatment by clinically comparing young (<56.6 years) and old (>71.3 years) patients with esophageal and esophagogastric-junction adenocarcinoma. Specifically, the study aimed to investigate the impact of age on the utilization and effectiveness of neoadjuvant treatment in these patient populations. In the methodology, patients with esophageal adenocarcinoma undergoing esophagectomy between 2001 and 2022 were retrospectively analyzed from three different centers. The study excluded patients with distant metastases or clinical UICC-stage I. The analysis utilized Cox proportional hazards regression to identify the variables associated with survival benefits. The key comparison was made between young and old patients, focusing on the administration of neoadjuvant treatment, survival outcomes, and histopathological regression. Through the analysis of 990 patients in different age groups who underwent neoadjuvant therapy or surgery alone, the authors found that older patients derive less benefit from neoadjuvant treatment in terms of OS improvement compared to younger patients. Additionally, older patients are more prone to side effects necessitating dose reduction, suggesting that upfront surgery may be a more suitable treatment approach for elderly individuals. They concluded the findings that neoadjuvant treatment was administered to a higher proportion of young patients compared to elderly patients. Young age was associated with a significant OS benefit after neoadjuvant treatment, with a median OS of 85.6 months versus 29.9 months for surgery alone. In contrast, elderly patients experienced a survival benefit equal to the length of neoadjuvant treatment itself, with no significant improvement in median OS. Despite differences in median OS benefit, histopathological regression was similar between the two age groups. Furthermore, it was observed that more elderly patients had a dose reduction or termination of neoadjuvant treatment, indicating a higher incidence of side effects in this group. As a conclusion, the study suggested that older patients benefit less from neoadjuvant treatment compared to younger patients in terms of gain in OS, and due to experiencing more side effects requiring dose reduction, upfront surgery should be considered as the primary treatment option in elderly patients. While the study by Rompen and colleagues is insightful, further discussion is warranted on certain aspects.

Firstly, the influence of neoadjuvant therapy on patients can lead to changes in their pathological stage, rendering it less reliable compared to the clinical stage, especially when comparing the p stage with the yp stage^2,3^. In older patients receiving neoadjuvant treatment, ~83% exhibit cT3 and 9% exhibit cT4, proportions significantly higher than those undergoing surgery alone. A similar trend is observed in the cN stage. This raises the question of whether older patients if matched for comparable characteristics, could potentially benefit more from neoadjuvant therapy.

Similarly, a comparable pattern was observed in the young patients group, revealing no notable advantage for patients undergoing neoadjuvant therapy in the radio-chemotherapy and other groups. Nonetheless, the table highlights a significant disparity in the clinical stages of patients receiving neoadjuvant therapy compared to those undergoing surgery alone, indicating a disadvantaged population in the neoadjuvant therapy group. Consequently, the outcomes of neoadjuvant chemoradiotherapy could potentially be influenced by bias.

Lastly, the principal aim of this study was to determine the advisability of neoadjuvant therapy for elderly patients with adenocarcinoma of the esophagus and gastroesophageal junction. The critical question is whether OS for elderly patients is improved by neoadjuvant therapy, rather than making comparisons between younger and older groups. The findings suggest that while neoadjuvant therapy yields similar survival rates to surgery alone, it also leads to more side effects necessitating the dose reduction. Consequently, the study recommends considering upfront surgery as the initial treatment strategy for elderly patients. However, it’s noteworthy that the neoadjuvant group presented with significantly more advanced disease stages than those in the surgery-alone group. This observation could imply, contrary to the study’s conclusions, a potential benefit of neoadjuvant therapy for elderly patients, particularly since it was associated with a marginally extended median overall survival in a cohort with more advanced disease despite the lack of statistical significance. Given this, further analysis is required to validate the original study’s conclusion.

In the realm of esophageal cancer treatment, surgical intervention remains the cornerstone, complemented by radiotherapy, chemotherapy, and increasingly, immunotherapy^4–7^. Both preoperative and postoperative treatments play a crucial role in influencing patient survival, with key surgical considerations including the extent of lymph node dissection, resection of the thoracic duct, and the choice of anastomosis sites^8–12^. These factors are closely linked to postoperative complications and overall survival rates. Recent studies suggest that even in elderly patients, the removal of more than 20 lymph nodes should be considered^12^. This emphasis on the elderly population has sparked a significant amount of research aimed at optimizing treatment approaches for this demographic, laying the groundwork for personalized treatment strategies. In 2023, a study investigated the efficacy of combining S-1-based chemoradiotherapy (CRTCT) with simultaneous integrated boost radiotherapy (SIB-RT) compared to SIB-RT alone in elderly patients (aged 70 years and older) with inoperable esophageal cancer. The results indicated a significant improvement in OS and progression-free survival (PFS) in patients receiving the combined treatment, without a notable increase in severe adverse effects. This suggests that S-1-based CRTCT could be a more effective and well-tolerated treatment option for this patient group, addressing the crucial need for personalized cancer therapies that take into account both effectiveness and quality of life, particularly in older individuals with potentially higher rates of comorbidities^13^. The choice of S-1 as the chemotherapeutic agent is noteworthy, as it is an orally administered drug that combines tegafur (a prodrug of 5-fluorouracil) with two modulators to enhance its antitumor effects and minimizes gastrointestinal toxicity, making it especially suitable for elderly patients who may not tolerate conventional intravenous chemotherapy regimens^13^.

Given the unique physiological characteristics and increased likelihood of comorbidities in elderly patients, the surgical approach, as a primary treatment modality, requires careful consideration of the patient’s overall health and ability to withstand surgery^12^. Evidence suggesting the beneficial impact of extensive lymph node dissection challenges traditional caution in subjecting older individuals to more extensive procedures, emphasizing the importance of a thorough evaluation of the patient’s condition and the potential long-term survival benefits^14–17^. Integrating radiotherapy, chemotherapy, and immunotherapy into the treatment regimen for elderly patients requires a delicate balance, considering potential side effects and overall treatment tolerance. Personalizing treatment through geriatric assessment tools and predictive markers could optimize outcomes by tailoring therapies to individual health status and tumor characteristics^13,17–20^. Recent research on elderly patients with esophageal cancer has provided valuable insights into treatment feasibility and outcomes, emphasizing the need for a multidisciplinary approach involving surgeons, medical oncologists, radiation oncologists, and geriatric specialists to develop a comprehensive and personalized treatment plan. In a phase 3 randomized clinical trial conducted in 2021, the efficacy and toxic effects of concurrent chemoradiotherapy (CCRT) with S-1 were compared to radiotherapy (RT) alone in older patients with esophageal cancer^20^. The study revealed that CCRT with S-1 was well-tolerated and provided significant benefits over RT alone in terms of OS and PFS. The primary endpoint, a 2-year overall survival rate, showed a marked improvement in the CCRT group compared to the RT alone group. Additionally, the CCRT group exhibited a higher complete response rate and better OS rates at 1, 2, and 3 years. These results indicate that CCRT with S-1 can effectively extend survival outcomes in older patients with esophageal cancer. In terms of safety, the incidence of grade 3 or higher toxic effects was similar between the CCRT and RT groups, except for a higher occurrence of grade 3 or higher leukopenia in the CCRT group. Treatment-related deaths were rare in both groups, with radiation-associated pneumonitis being the most common cause. Importantly, the overall tolerability of CCRT with S-1 suggests that this treatment regimen can be effectively managed in older patients. The treatment of esophageal cancer in elderly patients is a complex endeavor that requires a nuanced approach, balancing surgical intervention, including meticulous lymph node dissection, with the patient’s overall health and the integration of other treatment modalities. This collaborative effort is essential in navigating the complexities associated with treating this vulnerable population, aiming not only for survival but also for preserving the quality of life^13,18–23^.

In summary, the study by Rompen and colleagues offers a valuable contribution to the literature, providing evidence that could catalyze a shift towards age-specific treatment stratification in esophagus and gastroesophageal junction. Its findings have implications for the optimization of patient outcomes and the reduction of treatment-related morbidity in an elderly population that is often at a higher risk for both. While the study provides valuable insights, further discussion and analysis are warranted to address the potential biases in patient populations, the reliability of comparing clinical and pathological stages, and the potential benefits of neoadjuvant therapy for elderly patients. These considerations will contribute to a more comprehensive understanding of the age-dependent use and effectiveness of neoadjuvant treatment in patients with adenocarcinoma of the esophagus and gastroesophageal junction.

## Ethics statement

None.

## Source of funding

This work was supported by grants from the Scientific Research Fund of Education Department of Yunnan Province (2024J0261).

## Author contribution

All authors: study concept and design, acquisition, analysis, or interpretation of data. K.L.: drafting of the article and statistical analysis. T.K.: administrative, technical, or material support and Obtained funding.

## Conflicts of interest disclosure

The authors declare no conflicts of interest.

## Research registration unique identifying number (UIN)

None.

## Guarantor

Kexun Li and Tengfei Ke are guarantors.

## Data statement

This study does not involve new data, only data from the original article.

## References

[1] Rompen IF Crnovrsanin N Nienhuser H . Age-dependent benefit of neoadjuvant treatment in adenocarcinoma of the esophagus and gastroesophageal junction: a multicenter retrospective observational study of young versus old patients. Int J Surg 2023;109:3804–3814. 37720939 10.1097/JS9.0000000000000713 PMC10720874

[2] Shapiro J van Lanschot J Hulshof M . Neoadjuvant chemoradiotherapy plus surgery versus surgery alone for oesophageal or junctional cancer (CROSS): long-term results of a randomised controlled trial. Lancet Oncol 2015;16:1090–1098. 26254683 10.1016/S1470-2045(15)00040-6

[3] Yan X Duan H Ni Y . Tislelizumab combined with chemotherapy as neoadjuvant therapy for surgically resectable esophageal cancer: a prospective, single-arm, phase II study (TD-NICE). Int J Surg 2022;103:106680. 35595021 10.1016/j.ijsu.2022.106680

[4] Yang H Liu H Chen Y . Neoadjuvant chemoradiotherapy followed by surgery versus surgery alone for locally advanced squamous cell carcinoma of the esophagus (NEOCRTEC5010): a phase III multicenter, randomized, open-label clinical trial. J Clin Oncol 2018;36:2796–2803. 30089078 10.1200/JCO.2018.79.1483 PMC6145832

[5] Kelly RJ Ajani JA Kuzdzal J . Adjuvant nivolumab in resected esophageal or gastroesophageal junction cancer. New Engl J Med 2021;384:1191–1203. 33789008 10.1056/NEJMoa2032125

[6] Goto H Oshikiri T Kato T . The influence of preoperative smoking status on postoperative complications and long-term outcome following thoracoscopic esophagectomy in prone position for esophageal carcinoma. Ann Surg Oncol 2023;30:2202–2211. 36539581 10.1245/s10434-022-12898-y

[7] He W Leng X Mao T . Toripalimab plus paclitaxel and carboplatin as neoadjuvant therapy in locally advanced resectable esophageal squamous cell carcinoma. Oncologist 2022;27:e18–e28. 35305102 10.1093/oncolo/oyab011 PMC8842349

[8] Li K Leng X He W . Resected lymph nodes and survival of patients with esophageal squamous cell carcinoma: an observational study. Int J Surg 2023;109:2001–2009. 37222685 10.1097/JS9.0000000000000436 PMC10389544

[9] Fujisawa K Ohkura Y Ueno M . Nutritional outcomes of thoracic duct resection for radical esophagectomy by assessing body composition changes in one year: a single-center retrospective study. Ann Surg Oncol 2021;28:8414–8425. 34085142 10.1245/s10434-021-10222-8

[10] You J Zhang H Li W . Intrathoracic versus cervical anastomosis in esophagectomy for esophageal cancer: a meta-analysis of randomized controlled trials. Surgery 2022;172:575–583. 35400502 10.1016/j.surg.2022.03.006

[11] Li K Nie X Li C . Mapping of lymph node metastasis and efficacy index in thoracic esophageal squamous cell carcinoma: a large-scale retrospective analysis. Ann Surg Oncol 2023;30:5856–5865. 37227576 10.1245/s10434-023-13655-5

[12] Horikawa M Oshikiri T Kato T . Efficacy and postoperative outcomes of laparoscopic retrosternal route creation for the gastric conduit: propensity score-matched comparison to posterior mediastinal reconstruction. Ann Surg Oncol 2023;30:4044–4053. 37088861 10.1245/s10434-023-13345-2

[13] Li K Li C Nie X . Surgical vs nonsurgical treatment for esophageal squamous cell carcinoma in patients older than 70 years: a propensity score matching analysis. J Gastrointest Surg 2024;5:611–20. 10.1016/j.gassur.2024.01.010 38704198

[14] Wang X Han W Zhang W . Effectiveness of S-1-based chemoradiotherapy in patients 70 years and older with esophageal squamous cell carcinoma: a randomized clinical trial. Jama Netw Open 2023;6:e2312625. 37195667 10.1001/jamanetworkopen.2023.12625 PMC10193185

[15] Li K Leng X Peng L . Resected lymph nodes and survival of patients with esophageal squamous cell carcinoma in pT2 and pT3. Int J Surg 2024;110:1879–1881. 38116658 10.1097/JS9.0000000000001023 PMC10942199

[16] Florea IB Shersher DD . Do nodal disease patterns and approach to lymphadenectomy affect survival in patients with esophageal squamous cell carcinoma? reinvigorating an age-old debate. Ann Surg Oncol 2024;31:3584–3586. 38494561 10.1245/s10434-024-15176-1

[17] Altorki N Mynard N Nasar A . Ten-year survival and recurrence patterns after three-field lymph node dissection for squamous cell and adenocarcinoma of the esophagus. Ann Surg 2023;278:e43–e50. 35866662 10.1097/SLA.0000000000005627

[18] Li K Du K Liu K . Impact of two-field or three-field lymphadenectomy on overall survival in middle and lower thoracic esophageal squamous cell carcinoma: a single-center retrospective analysis. Oncol Lett 2023;25:189. 37065785 10.3892/ol.2023.13774 PMC10091183

[19] Yu Y Meng FY Wei X . Neoadjuvant chemotherapy combined with immunotherapy versus neoadjuvant chemoradiotherapy in patients with locally advanced esophageal squamous cell carcinoma. J Thorac Cardiov Sur 2024. 10.1016/j.jtcvs.2023.12.030 38246339

[20] Strickland MR Lander EM Gibson MK . Gastroesophageal adenocarcinomas with defective mismatch repair: current knowledge and clinical management. J Natl Compr Canc Ne 2024;22:1–8. 10.6004/jnccn.2023.7103 38503041

[21] Fick CN Dunne EG Sihag S . Immunotherapy for resectable locally advanced esophageal carcinoma. Ann Thorac Surg 2024. 10.1016/j.athoracsur.2024.02.021 PMC11194153 38408631

[22] Li K Du K Li C . Impact of metastatic lymph nodes on survival of patients with pn1-category esophageal squamous cell carcinoma: a long-term survival analysis. Ann Surg Oncol 2024;31:3794–3802. 38372864 10.1245/s10434-024-15019-z PMC11076366

[23] Ji Y Du X Zhu W . Efficacy of concurrent chemoradiotherapy with S-1 vs radiotherapy alone for older patients with esophageal cancer: a multicenter randomized phase 3 clinical trial. Jama Oncol 2021;7:1459–1466. 34351356 10.1001/jamaoncol.2021.2705 PMC8343504

